# Navigating causal reasoning in sustainability science

**DOI:** 10.1007/s13280-024-02047-y

**Published:** 2024-07-17

**Authors:** Maja Schlüter, Tilman Hertz, María Mancilla García, Thomas Banitz, Volker Grimm, Lars-Göran Johansson, Emilie Lindkvist, Rodrigo Martínez-Peña, Sonja Radosavljevic, Karl Wennberg, Petri Ylikoski

**Affiliations:** 1 Stockholm Resilience Centre, Stockholm University, Albanovägen 28, 114 19 Stockholm, Sweden; 2 Department of Ecological Modelling, Helmholtz Centre for Environmental Research – UFZ, Permoserstr. 15, 04318 Leipzig, Germany; 3 Department of Philosophy, University of Uppsala, Box 627, 751 26 Uppsala, Sweden; 4 The Institute for Analytical Sociology, Linköping University, 601 74 Norrköping, Sweden; 5 Department of Sociology, University of Helsinki, PO Box 18, 00014 Helsinki, Finland; 6 Stockholm School of Economics, Box 6501, 113 83 Stockholm, Sweden

**Keywords:** Accounts of causation, Causal analysis, Causal inquiry, Interdisciplinarity, Social–ecological systems

## Abstract

When reasoning about causes of sustainability problems and possible solutions, sustainability scientists rely on disciplinary-based understanding of cause–effect relations. These disciplinary assumptions enable and constrain how causal knowledge is generated, yet they are rarely made explicit. In a multidisciplinary field like sustainability science, lack of understanding differences in causal reasoning impedes our ability to address complex sustainability problems. To support navigating the diversity of causal reasoning, we articulate when and how during a research process researchers engage in causal reasoning and discuss four common ideas about causation that direct it. This articulation provides guidance for researchers to make their own assumptions and choices transparent and to interpret other researchers’ approaches. Understanding how causal claims are made and justified enables sustainability researchers to evaluate the diversity of causal claims, to build collaborations across disciplines, and to assess whether proposed solutions are suitable for a given problem.

## Introduction

When investigating the causes of sustainability problems and finding solutions to address them, sustainability scientists engage in causal reasoning. Scholars have, for example, put great efforts into identifying and quantifying the drivers of environmental change (e.g., IPBES et al. 2019; Tekwa et al. 2023), assessing the impacts of policies (e.g., Qiu et al. 2020), or causally explaining transformative change (Moore et al. 2014; Herrfahrdt-Pähle et al. 2020; Geels 2022). In their reasoning, researchers rely on disciplinary-based understanding of cause–effect relations. These conceptualizations stem from discipline-specific assumptions and methodologies, and they enable and constrain how knowledge of causation in social–ecological systems (SES) is acquired and used (Peters 2020; Banitz et al. 2022). The ideas and assumptions that inform causal reasoning are, however, rarely made explicit. While this is less problematic in disciplinary contexts with shared assumptions and norms, differences in causal reasoning and associated language can easily create barriers for communication, collaboration, and causal analysis in multi- or interdisciplinary contexts (Schlüter et al. 2023).

Sustainability science is a multidisciplinary field that involves researchers with backgrounds across the whole range of the natural and social sciences and the humanities (Kelly et al. 2019; Hazard et al. 2020; Pricope et al. 2020). It is thus not surprising that we find a diversity of ideas about causation, as well as strategies on how to make, justify, and communicate causal claims (Hertz et al. 2024). Some, for example, have put forward the application of statistical causal inference frameworks such as the potential outcomes framework (Ferraro et al. 2019), or novel data-driven methods (Sugihara et al. 2012; Runge et al. 2019). Others have pointed to emerging epistemologies of causation from the social sciences, such as analytical sociology (Biesbroek et al. 2017; Sieber et al. 2018), or political and historical sciences (Ermakoff 2019; Geels 2022). The causal inquiries of these studies have different goals, such as quantifying the effect of an intervention (Ferraro et al. 2019) or finding the causal mechanisms that explain an effect (Sieber et al. 2018). They employ different causal reasoning, address different causal questions, focus on different aspects of a SES, and have their own strengths and weaknesses. Consequently, the causal claims and their implications for understanding and intervening in SES may differ significantly.

Lack of understanding these differences and how they come about impedes our ability to address complex sustainability problems. First, there is no single research approach that can answer all causal questions about a complex problem and serve all research interests (Broadbent et al. 2016). If we don’t know the abilities of different approaches, we risk missing an opportunity to benefit from complementarities and finding the approach or combination of approaches that is most suitable for the problem of interest. Second, not understanding the reasoning that produced a causal insight impedes the wider use of the insight outside of the domain in which it was produced (Schlüter et al. 2023). Third, not knowing the assumptions that underlie a causal claim limits our ability to assess its scope and importance. This makes it difficult to achieve a more comprehensive understanding of the relations between the many diverse causes of sustainability problems (Banitz et al. 2022). Fourth, lack of awareness of epistemological differences makes it difficult to combine causal assumptions from different domains to model social–ecological interdependencies (Elsawah et al. 2020). And finally, the diversity of causal terminology associated with different ideas of causation, such as causal effect versus causal mechanism, has created confusion among sustainability researchers (Meyfroidt 2015).

Yet, despite increasing interest in causation in sustainability science, comprehensive guidance for navigating the diversity of causal reasoning is lacking (Colding and Barthel 2019). Understanding how different factors influence causal reasoning, such as the goals of an inquiry, fundamental ideas about causation, and research contexts, is crucial for interpreting researchers’ approaches, for evaluating the diversity of causal claims and the arguments that support them, and for assessing whether the proposed solutions are suitable for the problem at hand.

The aim of this paper is to help interdisciplinary sustainability scientists understand differences in causal reasoning and how these differences are, among others, the result of different ideas about causation. To this end, we (i) define causal reasoning and articulate when and how during a research process researchers engage in it, (ii) discuss four fundamental ideas about causation that direct causal reasoning, and (iii) illustrate the diversity of causal reasoning and its consequences for causal insights and action using examples from poverty studies. We draw on a long tradition of studying causation in philosophy of science (Woodward 2004; Beebee et al. 2009; Illari and Russo 2014), on ongoing discussions about causation in the social and natural sciences (e.g., Freese and Lutfey 2011; Walters and Vayda 2020; Geels 2022) and on insights from an interdisciplinary research environment in which all authors, with backgrounds in philosophy, physics, SES research, ecological modeling and sociology, have collaborated for five years. The awareness and understanding we aim to generate, together with our examples of how these differences may manifest in research and action, can help sustainability researchers critically reflect on the causal insights derived with a particular approach, to be more transparent about their own causal reasoning, to evaluate different kinds of causal claims, and to draw insights on cause–effect relations in complex SES in a better informed and robust manner.

## Causal reasoning

Causal reasoning consists of the cognitive activities we engage in when learning about the effects of manipulations, figuring out explanations, justifying assumptions of causal models or inferences, or attributing responsibility. It concerns the ways we think about causality and the considerations that enter into making causal claims (Illari and Russo 2014). Causal reasoning is fundamental to how we talk about, understand, study and act on the world (Sloman 2005; Gopnik and Schulz 2007; Lagnado 2021). We engage in causal reasoning for different purposes: inferring and describing causal relations (whether and to what degree does a cause lead to an effect?), intervention (what is the best way to bring about a desired effect?), prediction (what may happen in the future?), explanation (why and how did something happen?), or attribution of responsibility (what cause was decisive in bringing about an effect)? The purpose and the causal question direct the focus of the inquiry and influence what aspects of the causal setup of a phenomenon are considered. For example, if the goal is prediction, there is a strong focus on getting the outcome right, i.e., correctly assessing the magnitude of the causal effect, while much of the underlying causal process can be ignored. If the goal is explanation, however, the focus lies on the causal process, i.e., on how the cause or causes bring about the effect. If the goal is intervention, the focus of the causal inquiry is on those causes that can be modified, while other causes are ignored or relegated to background conditions.

**Fig. 1:**
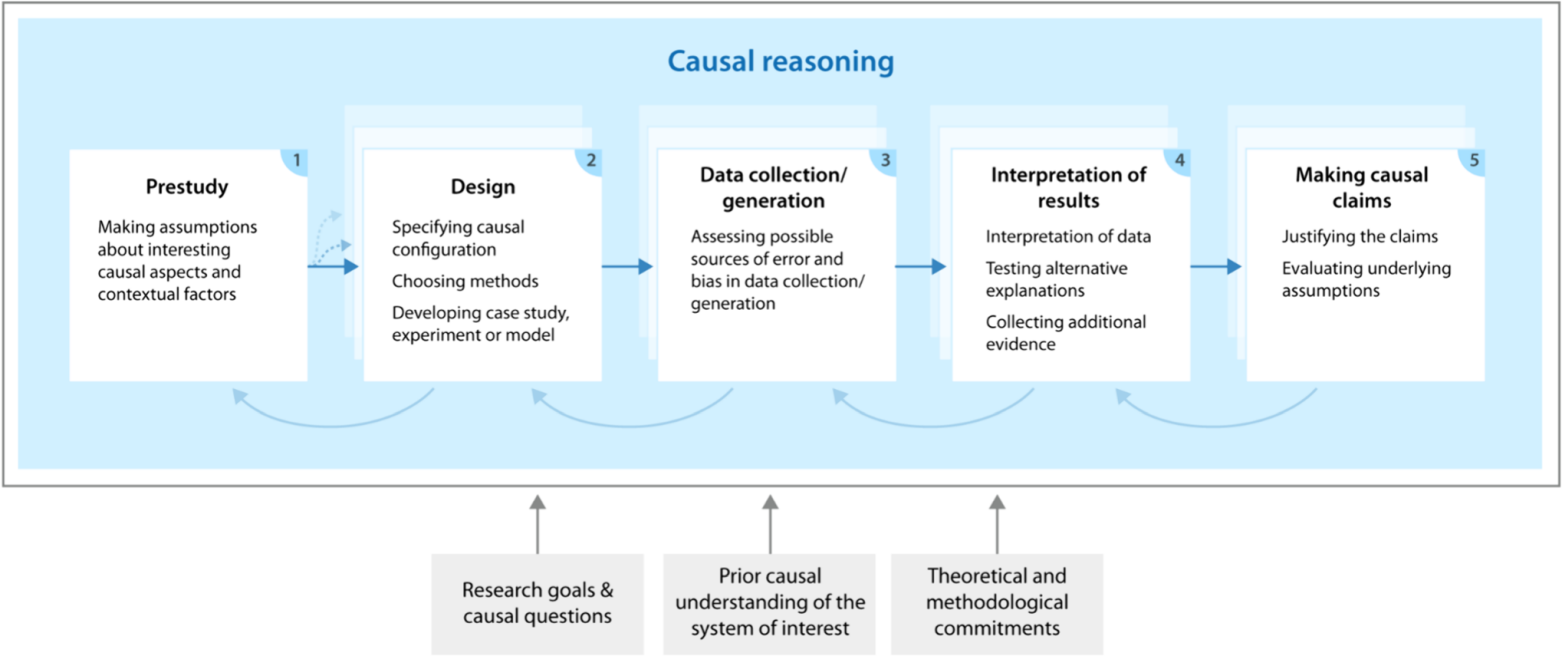
Different causal reasoning activities researchers engage in during the different phases of a research process

Causal reasoning takes place at all phases of a research process, but the activities and the elements that inform may be different in each phase (Fig. 1, Boxes 1–5). The process of causal reasoning is not linear, as choices in earlier phases may be influenced by later ones, e.g., concerns about how to interpret results may influence causal reasoning in the design phase. At the same time, choices early in the research process create a path dependence that influences causal reasoning later on (Fig. 1, shaded boxes and arrows between boxes).

At the beginning (Phase 1—Prestudy), researchers make assumptions about the broader context in which the causes and effects of interest operate and identify aspects and contextual factors or possible confounders that are important for the causal relations in focus. These assumptions and choices are influenced by the research goal, the research interest, and the underlying idea about causation (see accounts of causation in next section). They are also influenced by the broader research approach, including researchers’ theoretical and methodological commitments and background knowledge (Hertz et al. 2024), their prior experiences with and causal understanding of the system and phenomenon of interest as well as existing knowledge about the system and phenomenon (Fig. 1, gray boxes).

The design phase (Phase 2—Design) involves specifying the causal configuration of the phenomenon of interest, choosing appropriate methods for data collection or generation, and developing the research design. The choice of research design, e.g., a case study, an experimental or a model-based design, is informed by the goal of the study, the available background knowledge, preferences, theoretical and methodological commitments of the researchers, and scientific norms of the respective field. The choice of the design has important consequences for causal reasoning in subsequent phases of the research.

Causal reasoning during data collection or data generation involves assessing possible sources of errors and biases (Phase 3—Data collection/generation). This calls for constant vigilance because observations made during this phase might provide important cues about processes that may challenge the interpretation of the empirical results (Hand 2020). For example, for an experimental design it is important that the treatment and control units do not influence each other, i.e., that there are no spillovers which would make it difficult to evaluate the treatment effect (Ferraro et al. 2019; Schlüter et al. 2023). When choosing a case study design, a researcher will be concerned about possible biases in the selection of interview partners or other influences on the data collected during an interview.

Choices made during the design phase and possible errors and biases during data collection or generation influence the interpretation of results (Phase 4—Interpretation of results). Causal reasoning in this phase involves assessing evidence for causal relations or causal mechanisms and magnitudes of effects. The causal interpretation involves theoretical assumptions about causal relations that go beyond the empirical data. This phase also involves the consideration of alternative (causal) explanations for the observations. Often, to better justify the causal inferences of the study, some additional evidence is gathered. For example, evidence for a causal relation that has been identified through statistical analysis of observational data is strengthened through evidence about a mechanism that explains the relation (Bernedo Del Carpio et al. 2021). A causal mechanism that has been identified through process-tracing in a single case study (Bennett and Checkel 2014) is strengthened through reference to theory or mechanism schemes (Ylikoski 2018). Model-based claims about the causes of a system-level phenomenon are strengthened through testing alternative factors or processes through a sensitivity or robustness analysis (Grimm and Berger 2016).

Finally, when making causal claims (Phase 5—Making causal claims) a researcher engages in explicit justification trying to make the strongest possible case for the causal conclusions of the study and the causal assumptions underlying it. There are a variety of justificatory strategies (Peña et al. 2023) that may involve reference to the experimental design, to multiple forms of evidence for the claim, to the underlying theory, etc. The choice of justificatory strategy will depend on the employed research design and methods, e.g., the reasoning used to justify claims will differ considerably depending on whether they are based on an experimental study, a case study, or modeling.

## Basic ideas of causation

Among the theoretical commitments that shape causal reasoning are a researcher’s often implicit basic ideas about causation and how to study it. A basic idea about causation, also called an account of causation or a theory of causation, is a conceptualization of what constitutes a cause and an effect and the relation between them, as well as under which conditions and with what evidence we can make claims about cause–effect relations. There are many different accounts of causation, from generic ones that inform causal reasoning across a range of fields, to accounts that are specific to particular fields such as biology, behavioral sciences, or psychology (Illari and Russo 2014). We discuss four generic accounts because they are helpful starting points that cover many SES research approaches and have also been highlighted as fundamental accounts elsewhere (e.g., Brady 2011; Illari and Russo 2014). They are relatively well defined and distinguishable from each other and have either already been applied in SES research (the regularity and manipulability account), have recently received increased attention (the mechanism-based account), or have been proposed as a way to account for actor dependencies in complex systems (the intra-action-based account). While the first three are commonly discussed in philosophy of science (e.g., Brady 2011), the intra-action-based account is a recent development from process-relational philosophy based on the work of Karen Barad (Barad 2007). We include this account because of the growing interest in applying process-relational perspectives to SES research (Hertz et al. 2020; Mancilla García et al. 2020; West et al. 2020; Walsh et al. 2021), and because it raises interesting issues that are still debated in the philosophical literature.

In Table 1, we summarize key characteristics of the four accounts and list the methods and causal concepts that are commonly associated with them. Causal concepts are expressions that are used within a particular research context to describe causation. Some concepts and methods are specific to an account, and others are more broadly used.

**Table 1:** An overview of four common accounts of causation

	(1) Regularity account	(2) Manipulability account	(3) Mechanism-based account	(4) Intra-action-based account
Line of inquiry	Which later events are associated with the specified causes, or which earlier events are associated with the specified effects?	Does the effect change when the cause is manipulated?	How does the cause bring about the effect?	Why did a cause emerge in a particular way? How does this influence its effect?
Underlying basic idea of causation	Causation involves regular succession between types of events. The best way to learn about causal relations is to look for regularities between variables	Regular succession is not sufficient for causation; causal dependence can be best demonstrated with a controlled experiment. If we can produce B by producing A, A is a cause of B	Identifying regularities or causal dependencies is not enough to establish causation. We should also understand how the cause brings about the effect. If we understand the mechanisms, i.e., causal chains and configurations, that underlie observed or experimentally produced regularities, we can provide better explanations and better-justified causal claims	Understanding causation requires understanding why elements materialize the way they do. This draws attention to the material processes and discourses that together shape what elements are and what they can do. It also emphasizes that elements do not pre-exist an analysis but are constituted within the process
Key causal concepts	Independent variable, dependent variable, variance	Intervention, sensitivity, invariance, counterfactual dependency, confounders	Mechanism, causal process, generative mechanism, interdependent behavior, internal organization	Intra-action, process, relation, phenomena
Common methods	Multivariate statistical analysis	Randomized control trials, controlled experiments, observations about “natural experiments”	Multiple methods from (1) and (2), process-tracing, agent-based modeling	Multiple, often qualitative, methods that allow uncovering material-discursive practices
References	Suppes (1970), Hume (1981)	Pearl (2000), Holland (2001), Woodward (2006)	Craver (2007), Hedström and Ylikoski (2010)	Barad (2007)

### The regularity account

The regularity account of causation starts from David Hume’s observation that we learn about causation by observing how events regularly follow each other (within certain background conditions; Mill 1843; Mackie 1974; Hume 1981). Hume argued that causation consists of these observable regularities. Thus, there is no need to postulate hidden causal powers or obscure natural necessities, as was common among philosophers at the time. In more modern versions of the regularity theory, regularities are understood probabilistically (Suppes 1970): If A raises the probability of B occurring, it is a cause of B (given that confounders and covariates can be ruled out). The empiricists’ focus on observation, and a natural fit with the wide application of statistical methods in the sciences, positioned the regularity theory as the unofficial default view of causation for a long period of time. However, there are some long-standing problems: The regularity account has difficulty distinguishing between real causal relations and mere correlations produced by unobserved common causes (i.e., confounding variables). In addition, the regularity account can only be used when sufficient data are available and the relevant outcome variables have been identified. It thus requires the researcher to have a preconception of how the system works, including knowledge or assumptions about all potential causes and confounders.

The regularity account is most commonly used in data-intense, quantitative approaches, often in a predictive context. In SES research, Gutiérrez et al. (2011), for instance, identified fishery attributes that predict the success of co-management by analyzing community-level data from 130 co-managed fisheries. Using multivariate statistics and machine-learning techniques, they identified the presence of community leaders, strong social cohesion, individual and community quotas, and community-based protected areas as necessary conditions for successful co-management. Causal claims were justified through several criteria, such as the strength of association between the co-management attributes and success, the consistency of the association under various conditions, and temporality, where the presence of attributes preceded success. In addition, the authors derived support for their claims from assessing the plausibility of the causal explanation and the coherence with co-management theories and knowledge of each fishery. Thus, they combined insights from the regularity account with in-depth place-based knowledge and theories.

### The manipulability account

The manipulability account (Gasking 1955; Wright 1971; Woodward 2004) was developed to deal with the problem of confounding variables. Its basic idea is that if we can bring about B by bringing about A, A is a cause of B. This reasoning gives a neat and often practical way to sort out correlations between variables that are not causally linked. It is based on “counterfactuals,” i.e., propositions that tell us whether B would happen if the cause were different. For example, J’s drinking a lot of beer was a cause of them being drunk, because if we had prevented them from drinking, they would have stayed sober. As we did not limit their drinking, the conditional is a counterfactual.

From the point of view of the manipulability account, studies based on controlled experiments, rather than mere observations, are prototypical examples of scientific inquiry. To apply the manipulability account, the researcher needs access to data and a preconception of how the system works (Pearl and Mackenzie 2019)—just as they do with the regularity account. In addition, the researcher needs to have an opportunity to manipulate the target system or identify some valid source of externally imposed manipulation, such as a natural event or a policy measure. Another challenge is extending the approach beyond things that can be humanly manipulated, for example, to phenomena that are too distant, too large, change too slowly, or occurred in the past. Here the manipulationist must think about analogues and consider imaginary interventions, e.g., using thought experiments or modeling.

An example of the manipulability account of causation in SES is the use of controlled behavioral experiments to investigate how users of a common pool resource respond to a sudden drop in the supply of the resource when harvesting pressure exceeds a certain limit (Lindahl et al. 2016). Participants who played a game where the amount of resource changed abruptly after crossing a threshold (manipulation) cooperated significantly more, and under- or overexploited less, than if the resource decreased only gradually (control). Statistical analysis of the experimental data revealed that high resource-use efficiency was associated with cooperation and better knowledge of resource dynamics—and these, in turn, were associated with effective communication. The authors hypothesized that the threat of crossing a critical threshold triggers more effective communication, which enables better cooperation and knowledge-sharing, and thus more efficient resource use. However, extrapolating from controlled experimental settings is difficult, as there are many reasons why the observed effects might not occur in uncontrolled settings.

### The mechanism-based account

Neither the regularity nor the manipulability account reveals how a cause brings about its effect (Harré 1970; Elster 1989). This is the idea of the mechanism-based account. It focuses on the processes by which causal influence is transmitted from the cause to the effect. In this account, causation is not seen merely as a relation between variables, but a process about which we can make observations. The idea is that explanatory understanding comes from figuring out the mechanism, i.e., the set of entities, their properties, and activities, and how they are connected in time and space that are responsible for the phenomenon of interest (Craver 2007). The mechanism-based account of causation also helps to elucidate the relations between micro- and macro-scale processes, the role of heterogeneity in the environment and agents, and the contextual sensitivity of causal influence (Hedström and Ylikoski 2010).

The mechanism-based account of causation goes beyond observational data by putting stronger emphasis on theoretical thinking. The modern view of the mechanism-based account of causation has left behind the notions of determinism and reductionism to engage with stochasticity and levels of organization. Knowledge of causal mechanisms is also relevant for the justification of causal claims. Quite often, we have too little observational evidence and limited opportunities for experiments. Knowing about mechanisms can help us sort out spurious correlations and figure out the functional role of background conditions under which the observed causal regularities hold.

In the context of SES, Sieber et al. (2018) reconstructed the causal chains of events that explained why the governance of ecosystem-based adaptation reached an impasse in five case studies. They found six mechanisms—referred to as frame polarization, risk innovation, timing synchronization, rules of the game, lost in translation, and veto players—and described how they played out over time to create situations where it became increasingly difficult to overcome an impasse. The causal mechanisms emerge under specific contextual conditions and may interact with other mechanisms to generate the impasse. Frame polarization, for instance, refers to an interactive process in which the gap between decision-makers’ and stakeholders’ frames of what ecosystem-based adaptation is or should be rapidly increases until nobody is willing to discuss the content of the project any longer. The mechanism-based account helps to understand dynamic complexity as an explanatory cause and to move beyond static concepts such as barriers to change.

### The intra-action-based account

Process-relational philosophy unites a set of viewpoints that consider processes and relations as more fundamental than entities or actors (Whitehead 1929). Barad (2007) introduced the notion of “intra-action” to capture the idea that elements such as entities or actors do not exist as independent elements before they act on each other. Instead, they are constituted, or defined, through their intra-action, i.e., their constitution emerges from their relations. The account tracks these intra-actions to investigate what makes the elements involved in a causal relation be what they are in the first place. This is important, because what an element is impacts what causal influence it has.

To understand how relations constitute what an element is, Barad (2003) has put forward what she calls material-discursive practices. Material-discursive practices refer to how discourse and matter together produce elements in the world and shape what each and every element is and can do (Barad 2003, 2007; Orlikowski and Scott 2015; Neimanis et al. 2019; Hertz and Mancilla García 2021). These practices vary widely and are never finished. Material-discursive practices are not determined solely by human agency. Rather, humans and non-human elements are part of arrangements of discourse and matter that determine all elements, including humans (Bryant 2008). Consider, for example, the Andean concept of “ayllu.” In classical anthropology, it has been translated as a form of community organization involving collective property of land, organized collective work for common objectives and exchange of workforce among community members, thus separating “land” and “community” as two distinct and independent entities that could have different ways of relating or none at all, and still exist. Anthropologist Marisol de la Cadena (2015) interprets it from a relational perspective as a social–ecological practice that “takes place,” bringing together the people and the land in the Andes as one, existing only as they are together. Thinking of the ayllu in these terms mobilizes different causal capacities than conceiving it as a community owning land since this latter view separates society from nature. That is, what the ayllu is, the way it is defined and made sense of, determines the causal effects it has on other entities.

How matter and discourse are organized, i.e., relate to each other through their intra-actions, has thus relevance for causal reasoning. For example, a bottom trawling net only makes sense in a context in which the sea bottom (material dimension) is defined (discursive dimension) as a resource (Hertz and Mancilla García 2021). That is, the bottom trawling net emerges and causally affects organisms on the sea bottom through intra-action with the context that defines the sea bottom as a resource. These practices also intra-act with a wider set of notions such as productivity, optimality, etc. We could thus say that creating a bottom trawling net and considering that it makes sense to use it, enables these nets to affect the environment by reducing the fish population or affecting sea bottom habitats. Said otherwise, the discursive and the material are entangled in a particular organization of intra-active material-discursive practices that produce changes in the world.

In studying causation in SES, it is thus helpful to consider how specific contexts influence causal reasoning and explanations. Instead of seeing elements as stable, we recognize they are continuously shaped by material-discursive practices. This shifts focus from stability to a dynamic interaction between material and discursive factors, extending beyond social and ecological boundaries (Hertz and Mancilla García 2021). This approach helps us recognize what and how different properties are attributed to elements and what that produces. For instance, if a waterway is defined (discursive) and treated (material) as sacred rather than just a resource, it will lead to the waterway having different properties and thus different causal outcomes. Put differently, different material-discursive practices open up different properties which allow for different causal mechanisms to unfold.

The manipulability and the mechanism-based accounts were developed to address deficiencies of the regularity account. The manipulability account has been developed as a way to separate regularities that are based on a causal connection from those that are not. The mechanism-based account serves a similar purpose as the consideration of possible causal mechanisms underlying the observed regularity (and looking for evidence about their action) is a way to strengthen the credibility of a causal claim. Furthermore, the idea of a causal mechanism serves another purpose as it allows answering a follow-up question about the causal relation: How does the cause bring about the effect, thus expanding the causal explanation. The understanding of the underlying mechanism also provides important information about the conditions under which the observed causal regularity will hold and what kinds of things could modulate it. In practice, accounts are thus often combined in a process that successively increases the understanding about the causal relation of interest. In scientific studies, practices and argumentation do also often not strictly employ just one account, but become a mix influenced by the different elements of causal reasoning.

We now turn to a set of examples that illustrate how the use of different accounts of causation to study one specific problem provides very different insights into the causes of the problem and possible interventions for its alleviation.

## Studies of persistent poverty as illustrative examples of differences in causal reasoning

Persistent poverty is a pressing issue that has received attention across different research communities within sustainability science. Boxes 1, 2 and 3 describe three studies of poverty that pursue different goals and differ in their causal reasoning and resulting claims. The examples illustrate how the goals of a causal inquiry and underlying theoretical commitments shape causal reasoning. We can see how differences in causal reasoning produce different claims about the causes of poverty and strategies for its alleviation (Table 2). For example, the three studies draw on different types of system understanding to specify the causal configuration of interest in the design phase. The first study uses patterns between labor choice and poverty from a correlational study to select poor women’s labor choices as the causal variable that will be manipulated in an experimental study (Box 1). The second study uses assumptions about poverty–environment relations from the literature to model different types of contexts in which poverty trap dynamics may occur. The processes that generate trap dynamics are modeled following economic and resilience theory (Box 2). The third study develops a theoretical construct of a social–ecological trap based on theories of path dependence and traps from sociology and political sciences. The resulting ideal–typical structure of a trap is then tested in four case studies (Box 3).

**Table 2:** Overview of the goals and causal insights of the three studies. In the last column we draw conclusions about the implications of the given study for the design of poverty alleviation strategies (only studies 1 and 2 explicitly address implications for policy)

Example	Goal of causal inquiry	Accounts of causation	Claims about causal effect or explanation	Implications for poverty alleviation strategies
Bandiera et al. (2017)	To quantify the effect of an intervention to push poor households out of poverty	Manipulability account	Providing livestock and training to women in poor households caused a change in labor activities which resulted in an 21% increase in net annual earnings relative to control households	Poverty can be alleviated by providing individuals in households assets and the skills to use them
Lade et al. (2017)	To enhance understanding of how context influences the effectiveness of poverty alleviation strategies	Manipulability account Mechanism-based account	The effectiveness of a poverty alleviation strategy depends on the relation between agricultural practices and the biophysical environment in a given place. Neglecting these relations can reinforce poverty	Before intervening in a particular place it is important to consider the relation between agricultural practices and the social-ecological environment.
Boonstra and de Boer (2014)	To explain the emergence of poverty traps through historical and path- dependent processes	Mechanism-based account	A poverty trap emerges when a conjuncture of events triggers a path-dependent process and there are reinforcing processes that reproduce the trap	Structural conditions need to be changed so that a conjunction of social and environmental events that may produce trap processes cannot happen

**Box 1:** **Study 1—A randomized control trial to evaluate the effect of labor market choices on poverty in rural households in Bangladesh**

The goal of the study is to quantify the effect of an intervention to push poor households out of poverty through enabling the poorest women to take on the same work activities as better-off women in their villages (Bandiera et al. 2017). It is an example of causal reasoning in the context of statistical causal inference using an experimental approach and a difference-in-difference regression model. The authors first study household characteristics and the labor markets in villages in a region in Bangladesh. Using regression analysis, they show that there is a correlation between women’s type of labor activities and poverty, where poor women allocate most of their labor to low-return, casual jobs while richer women specialize in high-return livestock rearing. To assess the effect of changes in labor activities on poverty they conduct a randomized control trial (RCT), which builds on a manipulability account of causation. In the RCT, a cause, i.e., occupational choice of the ultra-poor, is manipulated through a treatment, i.e., a one-off provision of livestock assets and skills, to evaluate whether and to what extent the treatment changes the outcomes of interest, i.e., increases in earnings and diversification of the asset base which sets them on a sustained trajectory out of poverty. A positive effect of the treatment on the number of treated women switching to livestock rearing is thought to represent a mechanism to ensure a way out of poverty. Because the intervention provided both money and training it was, however, not possible to separate whether the cause for switching occupation was an increase in savings or skills In designing their study, the authors pay large attention to the process of randomizing the households selected for treatment and control. Randomization is important to control for any household differences that are not a result of the treatment. The quality of this randomization and the absence of spillover between treatment and control (Ferraro et al. 2019) are critical for the reliability of the causal inference, so researchers pay much attention to the design. The authors find evidence for their claim that reallocation of time from casual labor to livestock rearing leads to an increase in net annual earnings relative to control households by 21%. The account of causation and the theoretical commitments associated with its use in RCT direct the causal reasoning of this study (and others) to focus on properties of individuals that can be manipulated, such as access to skills or the means to purchase livestock. It thus neglects other structural influences on people’s (labor market) choices, such as gender and asset inequalities that influence and constrain agency in ways that perpetuate poverty (Akram-Lodhi 2020)

**Box 2:** **Study 2—A set of dynamical system models to assess the effectiveness of poverty alleviation strategies in different social–ecological contexts**

The goal of the study is to enhance understanding of how structural conditions, in particular the relations between poverty and state of the environment in rural contexts, influence the effectiveness of poverty alleviation strategies (Lade et al. 2017). It is an example of causal reasoning that employs a set of dynamical systems models to explore different what-if scenarios. The authors review commonly observed or assumed social–ecological relationships in local, agricultural, developing world contexts. They use this empirical knowledge and economic theory to develop a set of simple models of multi-dimensional poverty traps that incorporate different poverty–environment relationships, e.g., agricultural intensification that degrades the environment versus agricultural practices that maintain agroecological diversity. To assess the impact of these different contextual settings on the effectiveness of poverty alleviation strategies, they design a study that builds on the manipulability account of causation to assess the effects of interventions on poverty in different modeled contexts. The interventions, such as an asset input or a transformation in which farmers combine conventional agricultural production with traditional agricultural practices, are modeled through changes in initial conditions or structural changes in the model, respectively, and their effect on the emergence of non-poor system states is analyzed The authors build on economic theory that describes poverty traps systems with two equilibria, a poor and a well-being one, that are separated by a threshold (Barrett and Constas 2014). They pay much attention to extending this simple causal model by specifying different possible relations between financial, natural and cultural capital that are represented in the different model structures. Finally, they use resilience theory to inform the design of different types of interventions. Model results reveal that interventions such as asset inputs that ignore relations between agricultural production, nature and culture can, in some contexts, reinforce poverty. In such contexts, interventions that enable development while avoiding environmental and cultural degradation can help overcome traps. The causal claims are justified through systematically studying the connectivity between different factors and through contrasting the different models and interventions. In addition to manipulability, the study builds on the mechanism-based account of causation. Mechanism-based thinking directs attention to contextual factors, in this case the relations between agricultural practices and the environment, that influence what effect a particular cause has. It thus helps understand how contextual conditions may influence intervention effects, something that RCTs often do not explicitly take into account (Rodgers et al. 2020). However, the stylized nature of the models does not allow to quantify effects nor to apply them directly to a given case

**Box 3:** **Study 3—Historical studies to analyze the causal processes that created social–ecological traps**

The goal of the study is to understand the causal production of poverty and other types of traps through historical and path-dependent processes (Boonstra and de Boer 2014). It is an example of causal reasoning that uses single, historical case studies and a qualitative approach to test whether specified mechanisms produce traps. The authors draw on the concept of path dependence (Mahoney 2001) and previous work about the importance of timing (Mahoney and Rueschemeyer 2003) to investigate if and how the timing of historical events contributes to the emergence and persistence of social–ecological traps. They use an ideal–typical representation of a path-dependent process to analyze and systematically compare the historical sequence of ecological, economic and political events that trigger self-reinforcing feedbacks in four exemplary cases using the method of process-tracing The authors pay much attention to specifying the causal configuration, i.e., the ideal–typical structure of a social–ecological trap, which is then tested in the four cases. They find that all four cases exhibit the causal aspects that are typical for a path-dependent process, namely the conditions that trigger a path-dependent process (antecedent conditions and a critical juncture) and a process through which the trap reproduces itself (Boonstra and de Boer 2014). These results across the cases support their claim that historical processes and the timing of mutually interacting events are critical for producing trapped situations. The causal reasoning involved in this study is rooted in a mechanism-based account of causation and claims are justified through demonstrating the effect of critical junctures of events for triggering a trap across all four cases. The authors highlight the importance of systematically investigating why the trap solidified in a particular point in time and using counterfactual reasoning to justify this claim. Furthermore, they argue that the identification of causal mechanisms facilitates generalization beyond an individual case, which helps avoid idiosyncratic “just-so” stories. However, more research is needed to further generalize findings and assess the importance of other processes, such as human agency for the emergence of a trap (Boonstra and de Boer 2014). Furthermore, the long-time horizon of this historical study facilitates studying the role of structural factors, but less so the role of micro-level processes such as social actions of specific individuals	

Next to differences in how causes were identified, the studies also differed in how the effect was conceptualized. In the first one, the effect is a quantitative measure (% of increase in earnings relative to controls), in the second alternative states of the system (reaching a well-being state), in the third it is a process (the conjunction of events and the reproduction of the trap). Finally, all studies engage in some form of comparison to make their causal claims, although the reasoning involved when justifying causal claims differ. The first study compares households where the cause was manipulated with those where it was not and justifies claims through critically evaluating the experimental design and possible confounders. The second study compares the effect of a cause, i.e., an intervention, on poverty in different contexts and justifies claims through analysis of mathematical models of interdependencies; the third study compares critical junctures across cases to strengthen a claim about causal mechanisms. The examples also show that relying on the causal reasoning of only one research approach misses significant aspects of a complex SES problem, e.g., contextual (Study 2) or temporal mechanism (Study 3).

## Navigating the plurality of causal reasoning in sustainability science

### Making assumptions underlying causal reasoning transparent is important for communicating causal insights in multidisciplinary sustainability science

Much recent discussion about causation in SES has focused on methodological questions, while neglecting other important aspects of causal reasoning. Through expanding the scope of causal inquiry to go beyond questions of research design, methods, and interpretation of data, we get a broader view of differences between research approaches. Such a broader view is important because choices during all phases of a research process influence what causal knowledge is generated and hence what solutions are proposed. We encourage researchers to make their causal reasoning explicit by being transparent about the assumptions that direct choices in each phase of a causal inquiry. This is important for several reasons: First, it helps communicate causal insights to audiences that are not familiar with the hidden assumptions of the respective field. Second, being more precise about the context of a causal claim, such as the goal of an inquiry and background knowledge, can improve research designs, make a causal claim more understandable, and make people reflect why certain choices were made. Third, this will help sustainability scientists understand the scope of the claims of a study and assess whether the proposed solutions properly address the problem at hand. Finally, understanding what influences how causal claims are made facilitates interdisciplinary collaboration and integrating causal insights from the natural and social sciences in a social–ecological model.

Our articulation of the different causal reasoning activities and the elements that influence it provide guidance for sustainability researchers to explicate their own assumptions and choices and elicit or ask about those of others (Fig. 1; see also Hertz et al. 2024 for more examples and guidance on elements other than the accounts of causation that influence causal reasoning). Accounts of causation, such as the four highlighted here, are a good starting point to clarify differences and their consequences because they strongly influence how researchers conceptualize the relation between cause and effect and consequently seek evidence about it.

### Understanding differences between accounts of causation helps assess which approach or combination of approaches is suitable for a particular question and purpose

Different accounts of causation, both the ones highlighted here and others used in sustainability science, serve different goals and help answer different questions. In so doing, they afford different causal understanding. The manipulability account, for example, directs attention to causes that can be manipulated. Manipulability conditions can thus help us assess the effectiveness and efficiency of causal interventions in a complex system, but may miss unintended consequences and knowledge about the applicability of results to other contexts (geographic or in time) because specific contextual conditions are often neglected (Rao 2019). The mechanism-based account, on the contrary, focuses on multiple causes that are involved in causal chain or causal configuration, whether they are manipulable or not. Mechanism-based reasoning can help clarify under which conditions a particular outcome may be expected. Other accounts, such as the intra-action-based account, foreground contextual conditions for the emergence of causal influence. It helps understand how causal entities and processes are shaped in the first place. In our poverty examples, differences in the goals of inquiry and the associated causal reasoning have consequences for the resulting insights for poverty alleviation strategies: One study finds that household assets matter, the other that context matters, and the third points to the need to change contextual conditions to avoid trap-like dynamics (Table 2). The issue is thus to identify the type of question we ask, and the accounts, concepts or methods or combination thereof that serve best to answer the question.

The reasoning afforded by different accounts of causation provides argumentative support for a claim, but the types of evidence needed will be different, and they need to be logically consistent with an account’s principles. The regularity and manipulability accounts, for example, require large amounts of quantitative data and, in case of the latter, experimental designs which are often not feasible in SES research. At the same time there is much qualitative data which is not susceptible to statistical analysis but very relevant for causal analysis (Alexander et al. 2019). Finally, the accounts differ with respect to the degree to which they have been formalized. Formalization affects the ability for systematic testing of causal claims. For example, the regularity account can employ many statistical methods and it has formal language to talk about associations. Similarly, the potential outcomes framework or structured graphs, which are part of the manipulability account, provide a rigorous framework for designing and conducting a causal inference study. Other philosophical accounts have no mathematical framework to perform tests, therefore they have no formal language and little in the way of causal methods. However, the highly formalized tools are not sufficient to cover all causal reasoning involved in scientific inquiry, particularly in a multidisciplinary field such as sustainability science. This leaves room for other approaches. Some of them, such as the intra-action-based account or accounts rooted in the wider social sciences (see, e.g., suggestions in Geels 2022), are currently investigated for their use in sustainability science.

### Learning from differences and complementarities between research approaches improves the robustness of causal claims about sustainability problems

Each research approach has limitations when dealing with the complex, intertwined and multi-level nature of SES. Challenges of climate change, biodiversity decline, and ecosystem degradation, for example, occur at scales that are only partially amenable to the requirements of randomization in experimental, manipulability-based, approaches (Rodgers et al. 2020). Social–ecological dynamics may cause violations of key assumptions underlying statistical causal inference (Schlüter et al. 2023). Tracing mechanisms in single case studies requires a lot of data, some of which may be inaccessible, and justifying and generalizing causal claims from single case studies is challenging. Building on multiple approaches that differ in their causal reasoning can help overcome these limitations and improve causal inquiry (Antosz et al. 2022).

We take a pluralist stance that encourages mobilizing the plurality of causal reasoning in sustainability science in ways that do justice to the distinctness of each approach. Knowing the ideas of causation that shape causal reasoning is critical for making judgements if, when, and how different approaches may be combined and for which purpose. Reasoning based on the regularity, manipulability, and mechanism-based accounts, for instance, is often combined with the aim to strengthen a causal claim. For example, researchers may do an investigation based on the regularity account to assess whether a hypothesized cause regularly co-occurs with a hypothesized effect, as illustrated with our first poverty example. Subsequently, they may employ causal reasoning based on the manipulability or mechanism-based accounts to design a study to assess whether the observed regularity is the result of a causal relation between the variables. Causal reasoning patterns of various accounts are thus combined, yet their importance may vary during different phases of the research. A combination of different accounts of causation can also be found underlying the Bradford Hill criteria, which are commonly used in health sciences to infer a causal relation from a statistical association (Hill 1965).

There are other reasons one might consider working with different ideas about causation and the causal reasoning they afford, such as to uncover blind spots, generate new questions, or get a more complete picture of the multiple causes at play in a complex SES (Antosz et al. 2022; Schlüter et al. 2023; Hertz et al. 2024). Yet, the possibility to combine accounts is not without limitations and challenges (Banitz et al. 2022). Accounts may not be compatible since they build on different philosophical theories about the nature of causation, including distinct ideas about what is fundamental about causation. The intra-action-based account, for example, conveys a very different idea about what needs to be in focus when studying causation compared to the manipulability account. Instead of integrating approaches within one study, non-compatible accounts can be brought into dialogue in collaborative processes to support learning and critical reflections about the generated causal knowledge, which ultimately leads to a deeper, more nuanced and robust understanding of causation in SES.

## Conclusions

Knowledge about how to assess and distinguish the causal reasoning of a research approach can help evaluate causal claims and their justifications, and to find productive ways to develop areas of collaborative work that bring together the strengths of approaches and research designs, such as RCT and qualitative research designs (Rodgers et al. 2020), or process-tracing and agent-based modeling (Schlüter et al. 2023). The four basic ideas of causation and the five phases during a research process where causal reasoning takes place, provide a starting point for clarifying underlying assumptions. More reflection, transparency and interdisciplinary collaboration alone, however, is not sufficient to address complex sustainability challenges, which require processes that are embedded in and informed by collaborative and learning-oriented processes that also involve non-academic participants (Caniglia et al. 2020). Collaboration is thus needed not only between proponents of different approaches to causal analysis but also between those that advocate for more sophisticated causal inference methods and those that see solutions to problems as being about engagement, deliberation, and consultation rather than technical studies (Rodgers et al. 2020).
